# The menu varies with metabarcoding practices: A case study with the bat *Plecotus auritus*

**DOI:** 10.1371/journal.pone.0219135

**Published:** 2019-07-05

**Authors:** Tommy Andriollo, François Gillet, Johan R. Michaux, Manuel Ruedi

**Affiliations:** 1 Department of Mammalogy and Ornithology, Natural History Museum of Geneva, CP, CH, Geneva, Switzerland; 2 Section of Biology, Faculty of Sciences, University of Geneva, Quai Ernest-Ansermet, Geneva, Switzerland; 3 Laboratoire de Génétique de la Conservation, Université de Liège, Institut de Botanique B22, Liège, Belgium; 4 Comportement et Écologie de la Faune Sauvage (CEFS), Institut National de la Recherche Agronomique, Auzeville, Castanet-Tolosan Cedex, France; 5 CIRAD, Agirs Unit, TA C- 22/E- Campus international de Baillarguet, Montpellier Cedex, France; Stockholm University, SWEDEN

## Abstract

Metabarcoding of feces has revolutionized the knowledge of animal diets by providing unprecedented resolution of consumed resources. However, it is still unclear how different methodological approaches influence the ecological conclusions that can be drawn from such data. Here, we propose a critical evaluation of several data treatments on the inferred diet of the bat *Plecotus auritus* using guano regularly collected from various colonies throughout the entire active season. First and unlike previous claims, our data indicates that DNA extracted from large amounts of fecal material issued from guano accumulates yield broader taxonomic diversity of prey than smaller numbers of pellets would do, provided that extraction buffer volumes are adapted to such increased amounts of material. Second, trophic niche analyses based on prey occurrence data uncover strong seasonality in the bat’s diet and major differences among neighboring maternity colonies. Third, while the removal of rare prey items is not always warranted as it introduces biases affecting particularly samples with greater prey species richness. Fourth, examination of distinct taxonomic depths in diet analyses highlights different aspects of food consumption providing a better understanding of the consumer’s diet. Finally, the biologically meaningful patterns recovered with presence-absence approaches are virtually lost when attempting to quantify prey consumed using relative read abundances. Even in an ideal situation where reference barcodes are available for most potential prey species, inferring realistic patterns of prey consumption remains relatively challenging. Although best practice in metabarcoding analyses will depend on the aims of the study, several previous methodological recommendations seem unwarranted for studying such diverse diets as that of brown long-eared bats.

## Introduction

The advent of high-throughput sequencing (HTS) and metabarcoding approaches in particular provides unprecedented resolution in the study of animal diets [1–3]. Such sequencing techniques and related automated species identification allow the characterization of multiple assemblages of prey species through a single sequencing process, and has typically been applied to identify food resources recovered in feces, stomach contents or regurgitates [4]. So far, metabarcoding approaches has been used to unravel the diet of a diversity of invertebrates [5–7], fishes [8–10], reptiles [11], birds [12] or mammals [13–16]. It has been widely used in descriptive studies of diet composition, foraging strategies, and to resolve more complex questions about trophic ecology (e.g., resource partitioning, food web studies). These molecular techniques enable the study of elusive species’ diet such as that of insectivorous bats [17–25]. Before HTS, traditional methods of diet analysis of bat guano were based on morphological identification of macroscopic prey remains and relied on expert knowledge of invertebrate anatomy and diversity. Hence the taxonomic resolution was often limited to the order or family level [26–30]. With molecular methods of identification, the trophic ecology of these mammals can be characterized with much higher resolution across numerous samples [31–34], potentially unravelling overlooked dietary diversity, or identifying resource partitioning between species that was missed by traditional approaches [35].

Despite their huge promises, DNA-based methods are not free of limitations and potential methodological biases [36–39]. Experimental issues such as preferential amplification during PCR steps or the process of sequence demultiplexing and species identification have been examined in previous reviews [40–43]. However potential implications for a number of other factors that could lead to inaccurate diet analyses are still under explored. Here, we compare how some methodological choices may influence conclusions on dietary diversity and variations of trophic niche overlap in the diet of an insectivorous bat species, the brown long-eared bat *Plecotus auritus* (Linnaeus, 1758), which predominantly feeds on moths [28, 35]. Such a specialized diet offers ideal conditions for metabarcoding analyses as lepidopterans have been covered by global barcoding initiatives (see [44] and references therein), and thus relatively comprehensive reference databases exist for the molecular identification of most potential prey. Furthermore, the maternity colonies of long-eared bats are frequently established in buildings, allowing the easy collection of bat droppings without disturbing the animals.

Here, we monitored several maternity roosts established in close geographic proximity and throughout an entire period of occupancy to explore seasonal and inter-colonial variations in the exploited prey spectrum. The analyzed samples either included aggregates of few pellets (typically three) as recommended by Mata et al. [45], or larger pools of pellets (typically 15–20 pellets) in order to evaluate the diversity of prey consumed by animals from each maternity colony. As no bat was captured to avoid disturbances, we did not attempt to estimate individual-based diets, but only community or roost-level samples. Whole DNA extracts were subjected to classical metabarcoding approaches for molecular identification of prey, but the resulting original dataset was then altered in three different ways to see how such alterations would influence our conclusions about the long-eared bat’s diet. The alteration of the original dataset included (1) discarding rare prey items, (2) relying on a lower taxonomic depth and (3) quantifying prey contribution using sequence read counts. Indeed, removal of unique prey items from dataset is often recommended in metabarcoding studies, as rare items are purportedly more susceptible to reflect sequencing errors or may exaggerate their importance in diet diversity [36, 40]. However, no standard threshold has been established so far to define what a rare item is. Furthermore, the resulting effect of such removal on ecological conclusions still needs to be examined. The impact of the level of taxonomic resolution used for prey identification may also impact on ecological conclusions. This is especially likely when metabarcoding studies need to be compared with the ones leading to coarser identification levels, typically those using morphological identification of prey remains or those using barcoding markers with low taxonomic resolution. Finally, a recent study based on simulated datasets suggest that weighting prey occurrence according to read counts rather than simply recording their presence-absence may provide a more accurate view of consumers’ diet [38]. However results from these simulations have been poorly evaluated with real biological datasets that often include much more diversified diets.

## Material and methods

### Feces sampling and pooling

**Fig 1:**
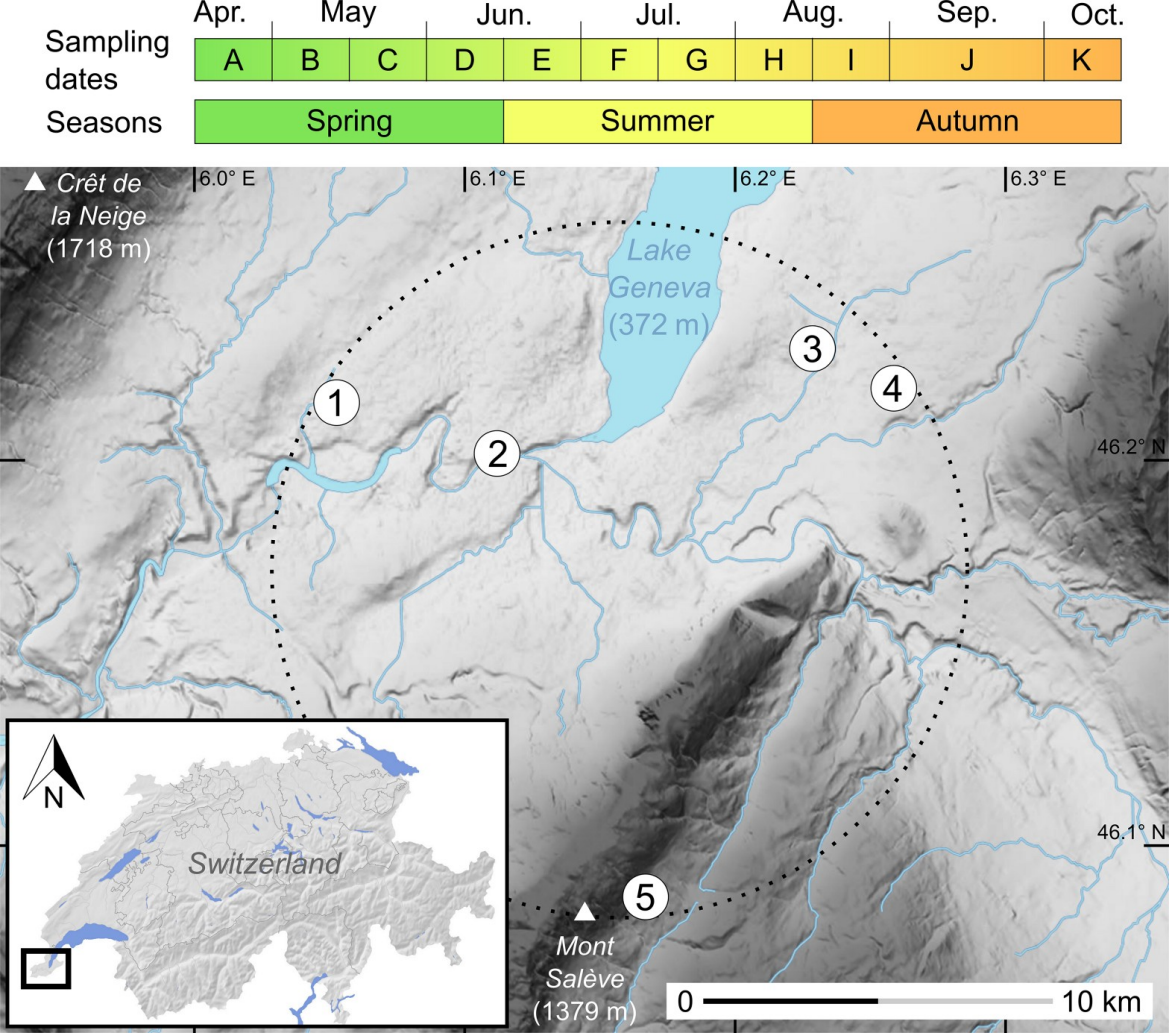
Sampling dates and map of the five colonies of long-eared bats studied in the Geneva region. Topographic slopes are shaded from pale (flat) to dark grey (steep). The dotted circle represents a virtual 10 km-radius area encompassing all the five sampled colonies: (1) Satigny, (2) pont Butin, (3) Choulex, (4) Presinge and (5) Sappey. The inset (lower left) provides a location map of the study area near Lake Geneva in southwestern Switzerland.

The sampling included feces collected from April to October 2015 from five monospecific colonies of genetically identified *Plecotus auritus* [46, 47]. These maternity colonies were situated in buildings (attics, steeples and a tunnel) and established within a 10 km-radius area in the Geneva region (Fig 1). They comprised a variable number of 10 to 60 individuals each. Four colonies were found in the lowlands at about 450 m a.s.l., while the fifth one was located in a more mountainous area at about 900 m a.s.l., but still in close geographic proximity to the others. In order to ensure that the fresh fecal material would dry quickly, thick absorbent paper sheets were set under hanging areas used by the bats. All accumulated feces were removed every two weeks (i.e. 11 dates in total), from the establishment of the maternity colony until bats eventually left the roost to complete their life cycle elsewhere. These sampling dates correspond to major periods in maternity roosts of bats [48], hereafter referred to as spring (from mid-April to mid-June, i.e. before pups are born), summer (from mid-June to mid-August, when pups are reared) and autumn (from mid-August to mid-October, when juveniles are weaned). The collected guano was stored in paper envelopes and preserved in a dry atmosphere before extraction.

For each colony and at each sampling date, we considered a “community sample” as a random aggregate of 15–20 pellets taken from the bulk of the collected droppings. These community samples weighted approximatively 60 ± 3 mg and likely represented the cumulated diet of the entire maternity colony during the two-weeks intervals, not the contribution of single individuals. In order to test whether smaller samples would yield an equivalent number of prey [45], we also sampled 6 independent biological replicates from each of the 11 collecting dates in the maternity colony of Satigny. In each of these replicates, hereafter called “small replicates”, only three pellets (about 8 mg) were randomly taken from the collected guano samples.

### Molecular analysis

DNA was extracted from each sample with the QIAamp DNA Stool Mini Kit (Qiagen, Switzerland) using protocol modifications suggested by Zeale et al. [49]. For the community samples, a further technical step was added to prevent the pipetting of too much fecal material into the centrifuge tube. We used two Eppendorf tubes instead of one filled with Buffer ASL; the guano samples were thus ground and soaked in twice the recommended volume of buffer before centrifugation. The supernatant of both tubes was then pooled for subsequent extraction steps. Purified DNA was preserved at -20°C. DNA extracts from all colonies and dates were randomized on plates to prevent artefactual colony or seasonal autocorrelation due to contamination between adjacent wells. To amplify a wide range of potential invertebrate prey, we used the primer pair ZBJ-ArtF1c and ZBJ-ArtR2c [49] which amplifies 157 bp of the COI barcode gene [50]. After library construction and equimolar multiplexing of purified PCR products, the final pool was sequenced on an Illumina Genome Analyzer II using 150 by 150 paired-end reads. Raw sequences were sorted and filtered using a script mixing FASTX Toolkit (http://hannonlab.cshl.edu/fastx/toolkit; 23-09-16) and USEARCH [51] functions as proposed by André et al. [52]. Briefly, the paired-end reads were joined on their overlapping ends. The overlap had to be at least 10 bases long with an 8% maximum difference. Primers were removed and sequences were filtered to keep only those with at least 90% of the bases with a quality index greater than Q30 [52]. DNA sequences shorter than 149 bp or represented by less than five reads were filtered out in order to remove likely sequencing errors. Within each sample, sequences represented by less than 0.1‰ of read counts were discarded in order to ensure evenness of sequencing depth across samples, and as recovery biases of this order of magnitude have been reported in metabarcoding analyses of mock communities [53]. The retained reads were clustered into unique molecular operational taxonomic units (MOTUs) using the software MEGAN [54], allowing for one mutation within each MOTU (Min Percent Identity: 99.0). MOTUs were then submitted to the NCBI BLAST tool [55] which relies on the GenBank database, and taxonomic identification from the resulting file was performed with MEGAN. The same MOTUs were independently identified through the BOLD sequence identification engine [56]; this taxonomically well-curated database allowed to gain taxonomic resolution for some MOTUs. The presence of blank extractions and PCR negative controls allowed us to exclude MOTUs likely originating from extraction or PCR contaminants from further analyses. A final taxonomic check was performed manually in order to ensure that each identified MOTU indeed corresponded to species known from inventories of invertebrates at local scale [57, 58] or across all Switzerland [59].

### Analyzed datasets

We followed Deagle et al. [38] to calculate the percentage of occurrence (POO) for each food item in the total dataset, weighted by the total number of prey found in a given sample (hereafter called dataset “wPOO”). This wPOO dataset was considered to be the original, unaltered dataset. We considered two altered versions of this wPOO dataset. Firstly, we removed all rare MOTUs, defined here as those found only in a single sample (dataset “No rare items”). Notice that such singletons were usually represented by high read numbers, so should not be assimilated to those MOTUs excluded from the raw database because they were represented by low read counts (see previous section). A second altered dataset was generated by retaining only the family of each identified prey to obtain a coarser level of taxon identification (dataset “Family level”). Finally, we also calculated the relative read abundance of each prey (dataset “RRA”), which assumes that the abundance of a prey is proportional to its sequence read counts. All data manipulations, computations, statistical tests and plotting were performed in R [60], using the packages dplyr [61], tidyr [62] and ggplot2 [63]. The effectiveness of sampling effort and that of sequencing depth per sample were analyzed using accumulation curves calculated with the package iNEXT [64]. The Chao2 minimum estimator of asymptotic species richness [65, 66] was computed with the software EstimateS 9.1.0 [67] for each sampling regime.

### Ecological indices

Trophic niche breadth for each community sample was calculated using the Levins’ index [68]. We calculated the seasonal niche breadth for each of the three periods considered (Fig 1), as the mean of Levins’ indices measured for all community samples in a given season. Departure from normality was assessed with Shapiro’s tests, and homogeneity (equality of variance) of indices was subsequently assessed either with Levene’s or F-tests, depending on the outcome of Shapiro’s tests. Statistical significance of differences in niche breadth was then tested by performing t-tests, accounting for differences in variance when necessary. For more detailed comparisons among colonies and across seasons, community samples from related dates were grouped within seasons (Fig 1). Trophic niche overlap between these seasonal samples was then measured with the Morisita-Horn index *C_λ_* [69, 70]. This measure is derived from the Simpsons diversity index and ranges from 0 (no overlap in utilization of resources) to 1 (complete overlap). Pairwise niche overlap constituted similarity matrices between samples of interest, and were graphically represented by multidimensional scaling (MDS), using the Principal Coordinates Analysis (PCoA) function implemented in the R package ade4 [71].

## Results

### Diet composition

The sequencing and initial sequence validation yielded to a total of 1973378 usable reads, or a mean of 16583 reads per sample (n = 119). These reads produced 881 distinct sequences that were clustered into 654 MOTUs, each represented by a 1125 read counts per sample on average (90% quantile: 5–5409). We discarded 111 of these MOTUs (17%) that did not match to any referenced sequence (i.e. with <40% similarity values). We also discarded 57 other MOTUs that obviously did not belong to the bat’s diet but likely resulted from environmental contamination. The sequences either did not represent animals (enterobacteriae, algae, fungi or rotifers), or were small arthropods known to be attracted or feeding on guano deposits (mites, machilids, anobiid and dermestid beetles). Finally, two species of slugs were also removed from the full dataset, as they likely represented secondary prey consumed by carabid beetles eaten by the long-eared bats. Extrapolation of species richness indicated that the sequencing of all samples was deep enough for detecting all prey species present in the sampled feces.

The final, complete dataset (used for wPOO and RRA) represented a panel of 521 identified arthropods consumed by brown long-eared bats in the Geneva region. These arthropods were classified into 3 classes, 15 orders and 94 families (S1 Table). 505 of these prey species (97%) were insects, 12 were spiders (2%), and the remaining 4 (1%) were woodlice. Among insects, 53% were lepidopterans (n = 269), 34% were flies (n = 173), while bugs, beetles, neuropterans and hymenopterans represented each 2% of consumed species. The other taxonomic groups (barklice, caddisflies, cockroaches, earwigs, orthopterans, scorpionflies and snakeflies) were only represented by less than five species. In addition to these well-identified arthropods, 63 MOTUs (12%) could not be reliably assigned to a species and were thus either kept identified to the family (n = 49) or to the order level (n = 14).

### Community samples vs. small replicates

**Fig 2:**
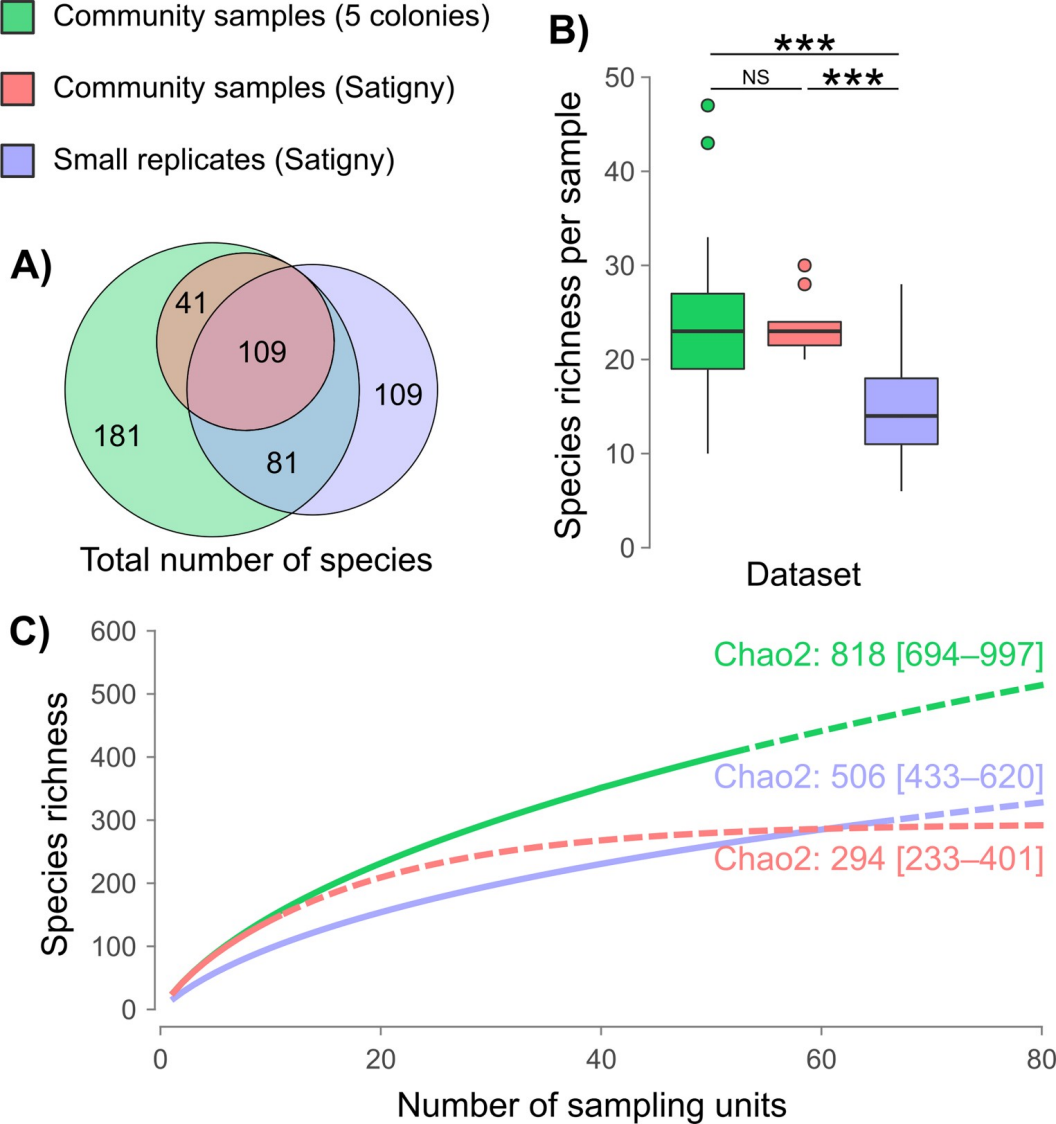
Species diversity statistics for different regimes of guano sampling: Community samples for all colonies in green (n = 53), community samples for the Satigny colony in red (n = 11), and small replicates in blue (n = 66). **A)** Area-proportional Euler diagram of the total species diversity found under each sampling regime. **B)** Number of species per sample, with significant differences between sampling regimes indicated by stars (*** P < 0.001). **C)** Extrapolated accumulation curves of the number of detected prey species for each sampling regime.

The 11 community samples and 66 small replicates collected in the maternity colony of Satigny produced a total 150 and 299 identifiable prey species, respectively, 109 of which were shared by both sampling regimes (Fig 2A). However, for a comparable sampling effort, community samples provided a significantly (P < 0.001) higher species richness (mean of 23.5 ± 3.1 prey species per sample) than did small replicates (14.6 ± 5.4; Fig 2B). When considering the 53 community samples collected in all five maternity colonies, the mean prey species richness (23.5 ± 7.1) was not different from that of Satigny only (Fig 2B). Extrapolations from accumulation curves further suggested that these numbers only represent 50 to 60% of potential prey richness inferred with Chao2 estimator (Fig 2C). These extrapolations also indicated that at least. 266 community samples would have been necessary to detect 95% of total species richness inferred for the five colonies (818 MOTUs).

### Seasonal variation of niche breadth

**Fig 3:**
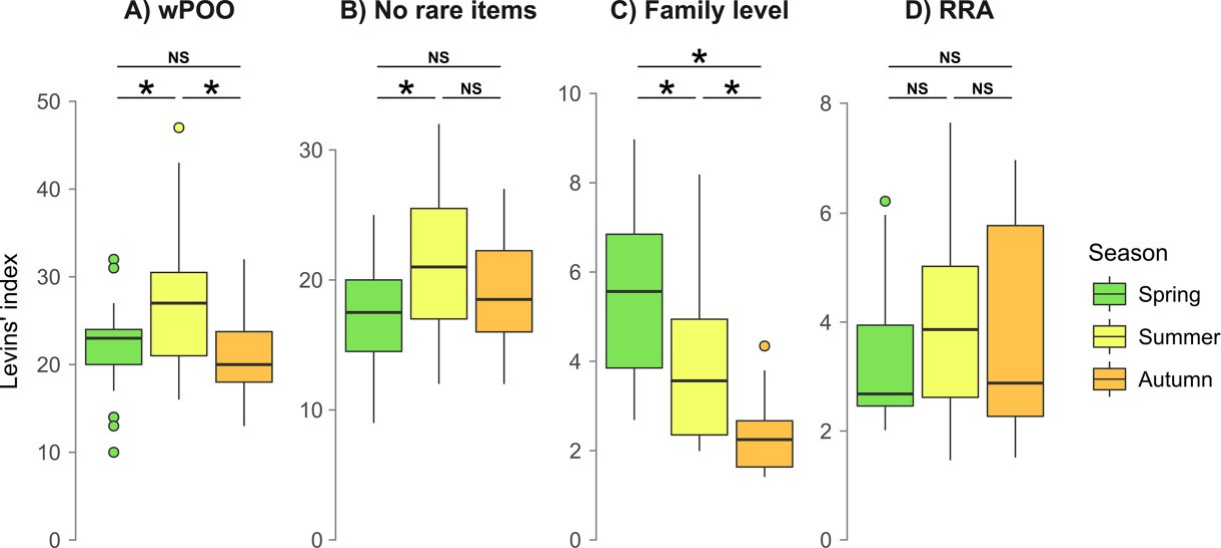
Seasonal trophic niche breadth variation (Levins’ index) measured in *P*. *auritus*. The four panels correspond to different data manipulations: **A)** full dataset, with all prey items kept and identified to the species level and considered as weighted occurrence data (wPOO); **B)** all unique occurrences discarded from the dataset (No rare items); **C)** prey identified to the family level only (Family level); **D)** all prey items weighted according to their relative read abundance (RRA). Significant differences are indicated by a star (P < 0.05).

Analysis of the complete dataset, comprising all retained prey items, each considered as weighted occurrence data (dataset wPOO), indicated that trophic niche breadth measured across all colonies was significantly higher in community samples gathered during the summer than during other seasons (P < 0.04) (Fig 3A). When expressed as number of prey species detected per community sample, a mean of 21.8 was observed in spring, 26.9 during the nursing season and 21.4 after reproduction. When rare prey species were removed (i.e. 232 MOTUs or 56% of all identified prey species), summer samples exhibited the highest niche breadth (21.7 prey species), which was significantly higher than during spring (16.8 prey species). Species richness of samples gathered in summer and autumn(18.9 prey species) did not differ significantly (Fig 3B). When using occurrences of prey identified at a coarser taxonomic resolution (dataset Family level), niche breadth statistically differed between all seasons, and continuously decreased throughout the year (Fig 3C). A mean of 5.6 families per sample were represented in spring, 4.0 families in summer, and 2.4 families in autumn, indicating a taxonomically more focused diet. Finally, when using the RRA dataset, no significant differences in trophic niche breadth were observed between seasons, and seasonal mean Levins’ measures ranged from 3.4 to 3.9 prey species (Fig 3D). Similar results were retrieved when using the Shannon-Wiener index of niche breadth [72], although it gives more weight to the rare resources (S1 File).

### Seasonal and geographic niche overlap

**Fig 4:**
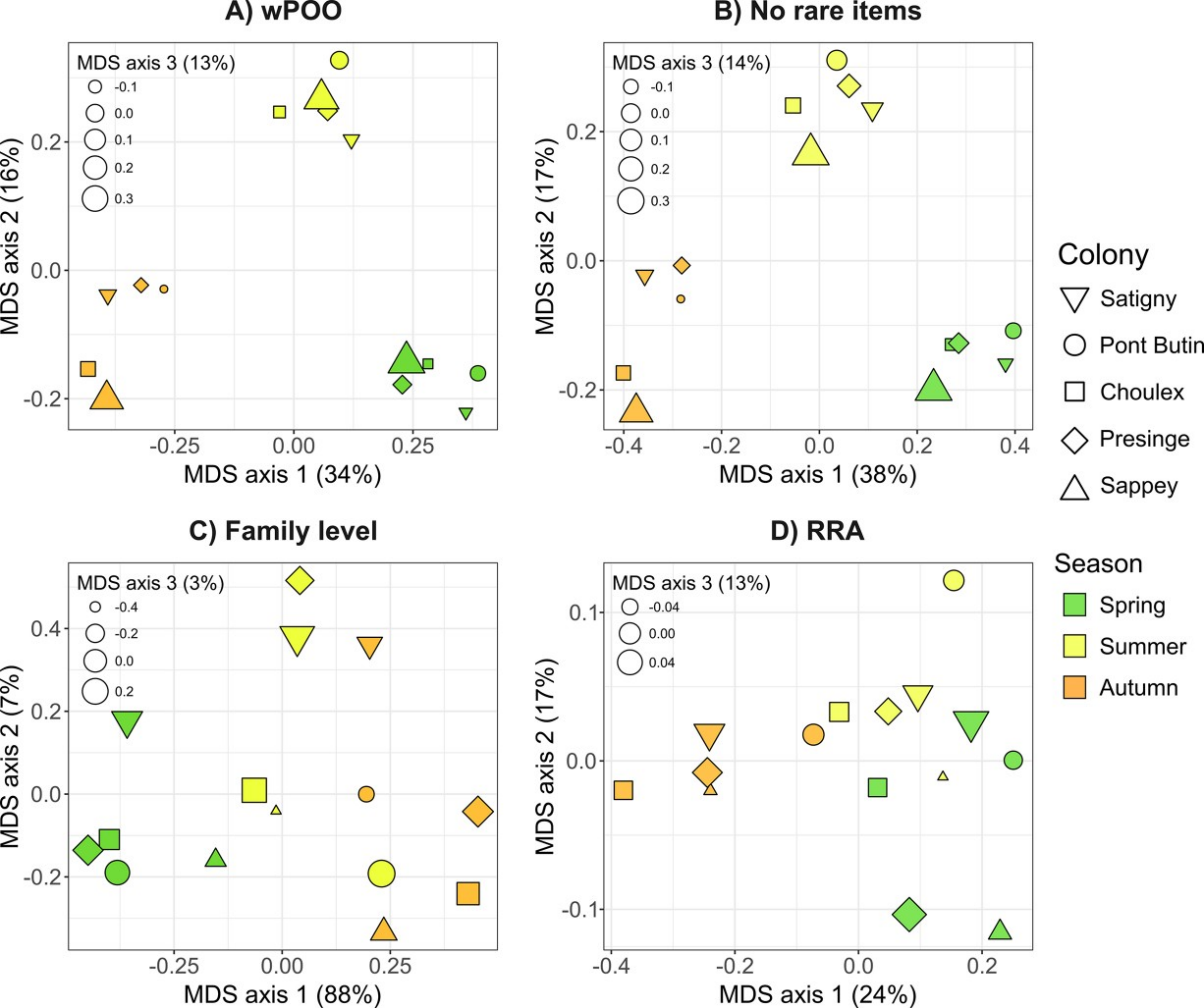
Multidimensional-scaling of trophic niche overlap (Morisita-Horn index) measured between fecal samples of *P*. *auritus* from different colonies and collected in distinct seasons. Each colony is represented by a distinct shape, and seasons by different colors. Size of symbols corresponds to their relative position along the third MDS axis. The four panels correspond to different data manipulations: **A)** full dataset, with all prey items kept and identified to the species level and considered as weighted occurrence data (wPOO); **B)** all unique occurrences discarded from the dataset (No rare items); **C)** prey identified to the family level only (Family level); **D)** all prey items weighted according to their relative read abundance (RRA).

In the similarity matrix calculated for the wPOO dataset, Morisita-Horn indices measuring trophic niche overlaps between maternity colonies and seasons ranged from 6 to 72%. Lowest overlap values were observed between spring and autumn (6–28%; mean 25%), while overlap values within seasons were systematically higher (28–72%; mean 47%), regardless of the colony considered. The Sappey colony consistently exhibited lower overlap measures with other colonies (28–52%; mean 38% within seasons), while all other colonies had larger overlaps (35–72%; mean 54% within season). This tendency was clearly recovered in MDS projection, since the three sampling seasons were well segregated along the first two axes of the PCA (Fig 4A). The third axis consistently separated the Sappey colony during all the three seasons considered (Fig 4A). The removal of rare prey items resulted in a very similar MDS representation (Fig 4B), and therefore did not affect the conclusions drawn from the full dataset. Conversely, both the use of family-level prey identification (Fig 4C) and the RRA method (Fig 4D) failed to provide a clear-cut segregation of samples from a given colony or of samples from a given season. When using such data alteration, samples were poorly discriminated by date on the first axis, and failed to identify the colony from Sappey as having lower dietary overlap compared to all other colonies. The use of taxonomic depth limited to the family level provided higher indices of niche overlap with most values being greater than 60%, while quantification by read abundance provided much lower values of this measure with most being lower than 20%. Comparable results were retrieved e,g, when using the Pianka’s *O_jk_* measure of niche overlap [73, 74] (S2 File).

## Discussion

### Prey detectability does not saturate in pooled poo samples

Mata et al. [45] compared the dietary diversity estimated for community samples against individual pellets, and observed no significant difference in the number of prey species detected under these two sampling regimes. They suggested that PCR competition between DNA templates could likely explain this saturation in prey detection, as DNA present in low frequency may be outcompeted by more abundant ones during amplification. They recommended avoiding pooling fecal samples to obtain a more accurate estimate of diet. We did not observe such saturation in our community samples consisting of 15–20 pellets versus smaller amounts of fecal material (3 pellets). Indeed, the former sampling regime allowed detection of 52% more prey species per sample than the latter (23.5 versus 14.9 prey species, respectively; Fig 2B). These results indicate that, given the same sequencing effort, the more pellets are pooled for the extraction, the higher diversity of prey species will be recovered. The apparent saturation in prey detection reported by Mata et al. [45] might therefore reflect a methodological problem during the extraction step (e.g., clogged membrane) rather than an amplification bias during PCR. Our extraction improvement consisted of using larger initial volumes of digestion buffer when extracting large volumes of fecal material and probably overcame this problem. Contrary to recommendation from Mata et al. [45], we thus suggest that community samples uncover greater dietary diversities than individual samples do. Furthermore, such community samples include droppings accumulated during several days and by several bats and thus better reflect the range of prey species consumed by the colony members. They also reduce the stochasticity associated with smaller samples, that are more affected by individual variation of prey consumption (e.g., [45, 75, 76]).

### Seasonal and geographical variation matters

Due to the marked seasonal phenologies of insect activity observed in most habitats [77], prey assemblages available for insectivorous bats varies greatly throughout the year, in terms of abundance, diversity and composition. With the complete dataset (wPOO; Fig 3A), we showed that this seasonality is strongly reflected in the diet of *P*. *auritus*, which appears to exploit insect prey opportunistically, with a peak in prey species richness marked during the summer, when females are rearing their pups. Members of all maternity colonies appear to exploit the same seasonal spectrum of insects, as niche overlap is much larger within a given season than between different periods (Fig 4A). These seasonal shifts in prey consumption imply that dietary habits measured at a given period may poorly reflect the global diet, both in terms of trophic niche breadth and composition, as already evidenced in other bats from temperate zones [19, 35]. Hence, studies focusing on temporarily limited samplings that are typically conducted during the summer (e.g., [78]; i.e. when prey availability may not represent limiting trophic resources) could miss crucial information about diet composition, or niche partitioning.

Despite the small geographic scale envisioned here (i.e., within a radius of 10 km; Fig 1), our data also shows that geographic location can be an important factor of variation. Indeed, the four colonies of brown long-eared bats from the lowlands had systematically higher niche overlaps among them as opposed to the one located higher in the mountains (Fig 4A). Such local effects of diet composition may thus confound effects of diet variation, when trophic niche overlaps are evaluated in distant localities (e.g., [28, 35]). To avoid these potential confounding factors of variation (season and location), we argue that niche overlap should be assessed using populations or species sampled in close proximity and within the same period of the year.

### Discarding rare prey occurrences is not always warranted

Rare prey items are sometimes removed from metabarcoding datasets, since they may have an exaggerated weight in analyses compared to their actual importance in the diet [21, 40], especially for animals consuming relatively few prey species [38]. However, this is not always warranted, as we show here with the highly diversified diet of the brown long-eared bat. In this case, removing rare occurrences, which represent more than half of all prey items recovered, did not notably affect indices of niche overlap (about 5% of increase), nor results concerning seasonal and geographic variation of diet (Fig 4B). Thus, we concur with Clare et al. [40] that discarding unique occurrences has little effect regarding some ecological conclusions, but would restrict this recommendation to the estimation of resource partitioning. Indeed, we show here that removing these rare items may lead to a significant shrinking of other measures such as indices of niche breadth. Since samples with the highest prey species richness also are the ones most susceptible to carry rare prey items, removal of the latter artificially increases similarities of niche breadths (Fig 3B).

In other situations where reference databases for taxonomic identification are incomplete (e.g., [79]) and rare MOTUs are difficult to tease apart from artefactual sequences [36], the removal of rare items is justified. In more ideal conditions, like shown here, these rare prey sequences can be easily and unambiguously assigned to plausible insect species and thus reflect real prey choices. The high number of unique prey occurrences observed throughout the year indeed reflects the opportunistic feeding behavior of the brown long-eared bat reported in other studies [29, 80]. These prey items should therefore be retained for an accurate description of the richness of the diet of this insectivore.

### Different taxonomic depth leads to different conclusions

The level of taxonomic resolution in prey consumed is known to greatly affect ecological analyses, such as food web connectance [81]. Taxonomic resolution is particularly limited in the case of diet studies using morphological identification of prey remains, but is also limited in molecular studies when reference databases are incomplete or when conserved markers are used in an attempt to reduce amplification biases. In the last case for instance, less than 67% of sequences were identified to the genus level and less than 30% to the species level in recent studies [82, 83]. In the context of dietary assessment, an increase in niche breadth and a decrease in niche overlap are expected when taxonomic resolution increases [35]. Accordingly, we observed that the trophic niche overlap between colonies of brown long-eared bats was clearly higher (by about 80%) when only taxonomic identifications to the order-level were considered (Fig 3A, Fig 3C). Moreover, this overestimation due to coarser taxonomic identification of prey systematically led to increased niche overlap between seasons. This overlap was even higher than the 60% threshold classically used to characterize strong dietary overlaps [84]. When using a better taxonomic resolution (species level), all indices were lower than 49% (mean 25%) and we would reach the opposite conclusion. The study of dietary overlap among colonies and seasons was also severely affected by the use of lower taxonomic depth, as both season- and colony-specific differences were much less apparent or lost (Fig 4C). This increase in values of niche overlap was probably exacerbated by the fact that *P*. *auritus* is a moth-specialist, and less dramatic effects can be expected when studying species with wider taxonomic dietary breadth. Still, when coarse levels of prey identification fail to reveal trophic resource partitioning between species (e.g., [85, 86]), metabarcoding techniques with high-resolution markers might be useful before rejecting the potential for competitive exclusion and invoking stabilizing mechanisms for coexistence.

Both detailed and coarser levels of taxonomic depths of prey identification might, however, highlight different aspects of food exploitation by insectivorous bats. The fully resolved dataset (Fig 3A) indicated that the brown long-eared bat exploits significantly higher prey species richness during the summer, but when comparisons were restricted to family level only (Fig 3C) a higher diversity of insects was consumed in spring. Although less numerous in terms of species richness, the spring prey insects represent a broader spectrum of families, suggesting that bats cannot rely on a few preferred taxonomic groups (e.g., the largest or the most profitable prey such as noctuid or geometrid moths), but must be more eclectic during this season. The constant decrease of taxonomic diversity of preys observed at the family level throughout the year (Fig 3C) might again be a sign of opportunistic feeding behavior of the brown long-eared bat, which is known to exploit the peaks of moth diversity and abundance in July-August [29]. This hypothesis, however, should be tested properly with feeding choices in order to be validated.

### When weighted occurrences perform better than relative read abundance

Comparisons of data treatments (Fig 4) showed that using quantitative methods based on sequence read counts (RRA) had also a dramatic effect on patterns of diet variation. Accounting for read abundance (Fig 4D) completely blurred the strong seasonal and geographical signature recovered with the wPOO approach (Fig 4A). Deagle et al. [38] suggested RRA approaches provide more accurate view of consumer’s diet when moderate amplification and recovery biases are present in the metabarcoding process. To show this, they simulated in silico biases ranging from 4× to 20× relatively to a standard amplification. Several lines of evidence indicate that much higher levels of recovery biases might actually occur in real metabarcoding analyses (up to 5000×[53]), and could therefore explain the poor performance of RRA in the case shown here (Fig 3, Fig 4). First, very few prey species received high relative read abundance, while most others were represented by extremely low values (<1‰; see S3 File), drastically downsizing the importance of the latter in measurements of niche overlap. Furthermore, several other potential biases due to the prey composition itself (e.g., presence of eggs) or to its digestibility certainly also influence the final outcome of read counts and can hardly be accounted for [34, 36, 87]. Other approaches, unexplored here, such as the use of multiple primer pairs [41, 88], the use of primers known to provide quantitative results [37, 43] or avoiding the PCR step by doing shotgun sequencing [89, 90] may be used to overcome part of the mentioned biases. It is also possible that composition of simpler diets may be better estimated by RRA than in the situation here [91, 92].

## Supporting information

S1 Table Taxonomic list of the 521 MOTUs recovered in *P. auritus* guano samples. (DOCX)

S1 File Seasonal trophic niche breadth variation (Shannon-Wiener index) measured in *P. auritus*. (DOCX)

S2 File Multidimensional-scaling of trophic niche overlap (Pianka’s O_jk_ index) measured between fecal samples of *P. auritus* from different colonies and collected in distinct seasons. (DOCX)

S3 File Impact of read numbers on frequency estimates. (DOCX)
