# Supplementary material for: The menu varies with metabarcoding practices: A case study with the bat Plecotus auritus
Source: PLoS One. 2019 Jul 5;14(7):e0219135. doi: 10.1371/journal.pone.0219135 (PMC6611578; doi:10.1371/journal.pone.0219135)
Supplement: S1 Table — (DOCX) [file pone.0219135.s001.docx]

# S1 Table. Taxonomic list of the 521 MOTUs recovered in *P. auritus* guano samples.

| **Class** | **Order** | **Family** | **MOTU** |
| --- | --- | --- | --- |
| Arachnida | Araneae | Agelenidae | *Coelotes terrestris* |
|  |  | Amaurobiidae | *Amaurobius fenestralis* |
|  |  | Anyphaenidae | *Anyphaena accentuata* |
|  |  | Araneidae | *Gibbaranea gibbosa* |
|  |  |  | *Nuctenea umbratica* |
|  |  | Clubionidae | *Clubiona corticalis* |
|  |  |  | *Clubiona pallidula* |
|  |  | Eutichuridae | *Cheiracanthium mildei* |
|  |  | Philodromidae | *Philodromus margaritatus* |
|  |  | Salticidae | *Salticus zebraneus* |
|  |  | Thomisidae | *Diaea dorsata* |
|  |  |  | *Xysticus lanio* |
| Insecta | Blattodea | Ectobiidae | *Ectobius sylvestris* |
|  |  |  | *Ectobius vittiventris* |
|  | Coleoptera | Carabidae | *Amara similata* |
|  |  |  | *Bembidion quadrimaculatum* |
|  |  | Cerambycidae | *Mesosa nebulosa* |
|  |  | Cleridae | *Opilo mollis* |
|  |  | Curculionidae | *Curculio glandium* |
|  |  |  | *Dorytomus longimanus* |
|  |  | Melyridae | *Dasytes aeratus* |
|  |  | Scarabaeidae | *Amphimallon majale* |
|  |  |  | *Rhizotrogus aestivus* |
|  |  |  | *Serica brunnea* |
|  |  | Staphylinidae | *Eusphalerum signatum* |
|  |  | Unknown | Coleoptera sp. |
|  | Dermaptera | Forficulidae | *Chelidurella guentheri* |
|  |  |  | *Forficula auricularia* |
|  | Diptera | Anisopodidae | *Sylvicola cinctus* |
|  |  |  | *Sylvicola* sp. |
|  |  | Anthomyiidae | *Botanophila fugax* |
|  |  |  | *Delia florilega* |
|  |  |  | *Delia platura* |
|  |  |  | *Delia radicum* |
|  |  |  | *Hydrophoria silvicola* |
|  |  |  | *Pegoplata infirma* |
|  |  |  | Anthomyiidae sp. 1 |
|  |  |  | Anthomyiidae sp. 2 |
|  |  | Bibionidae | *Dilophus febrilis* |
|  |  | Calliphoridae | *Bellardia vulgaris* |
|  |  |  | *Bellardia* sp. |
|  |  |  | *Calliphora vicina* |
|  |  |  | *Calliphora vomitoria* |
|  |  |  | Calliphoridae sp. |
|  |  |  | *Lucilia caesar* |
|  |  |  | *Lucilia sericata* |
|  |  |  | *Pollenia hungarica* |
|  |  |  | *Pollenia pediculata* |
|  |  |  | *Pollenia rudis* |
|  |  |  | *Pollenia vagabunda* |
|  |  | Cecidomyiidae | *Mayetiola destructor* |
|  |  |  | *Asteromyia* sp. |
|  |  |  | Cecidomyiidae sp. 1 |
|  |  |  | Cecidomyiidae sp. 2 |
|  |  |  | Cecidomyiidae sp. 3 |
|  |  | Ceratopogonidae | *Culicoides chiopterus* |
|  |  | Chironomidae | *Conchapelopia melanops* |
|  |  |  | *Paratrichocladius rufiventris* |
|  |  |  | *Polypedilum convictum* |
|  |  |  | *Polypedilum* sp. |
|  |  |  | Chironomidae sp. |
|  |  | Culicidae | *Culex pipiens* |
|  |  | Drosophilidae | *Drosophila melanogaster* |
|  |  |  | *Drosophila subobscura* |
|  |  |  | *Drosophila suzukii* |
|  |  |  | *Phortica* sp. |
|  |  | Empididae | *Empis bicuspidata* |
|  |  |  | *Empis chioptera* |
|  |  |  | *Empis ciliata* |
|  |  |  | *Empis stercorea* |
|  |  |  | *Empis tessellata* |
|  |  |  | *Empis trigramma* |
|  |  |  | *Empis variegata* |
|  |  |  | *Rhamphomyia crassirostris* |
|  |  |  | Empididae sp. 2 |
|  |  |  | Empididae sp. 3 |
|  |  |  | Empididae sp. 4 |
|  |  |  | Empididae sp. 5 |
|  |  |  | Empididae sp. 6 |
|  |  | Fanniidae | *Fannia pallitibia* |
|  |  |  | *Fannia polychaeta* |
|  |  |  | *Fannia scalaris* |
|  |  |  | Fanniidae sp. |
|  |  | Heleomyzidae | Heleomyzidae sp. |
|  |  | Lauxaniidae | Lauxaniidae sp. 1 |
|  |  |  | Lauxaniidae sp. 2 |
|  |  | Limoniidae | *Antocha vitripennis* |
|  |  |  | *Cheilotrichia cinerascens* |
|  |  |  | *Dicranomyia tristis* |
|  |  |  | *Limonia nigropunctata* |
|  |  |  | *Limonia nubeculosa* |
|  |  |  | *Metalimnobia bifasciata* |
|  |  |  | *Rhipidia maculata* |
|  |  | Lonchaeidae | Lonchaeidae sp. |
|  |  | Muscidae | *Hebecnema fumosa* |
|  |  |  | *Helina abdominalis* |
|  |  |  | *Helina evecta* |
|  |  |  | *Helina impuncta* |
|  |  |  | *Helina lasiophthalma* |
|  |  |  | *Helina reversio* |
|  |  |  | *Helina* sp. 1 |
|  |  |  | *Helina* sp. 2 |
|  |  |  | *Hydrotaea irritans* |
|  |  |  | *Musca autumnalis* |
|  |  |  | *Muscina levida* |
|  |  |  | *Muscina pascuorum* |
|  |  |  | *Muscina prolapsa* |
|  |  |  | *Phaonia errans* |
|  |  |  | *Phaonia pallida* |
|  |  |  | *Phaonia rufiventris* |
|  |  |  | *Phaonia subventa* |
|  |  |  | *Phaonia trimaculata* |
|  |  |  | *Phaonia tuguriorum* |
|  |  |  | *Phaonia* sp. |
|  |  |  | *Polietes lardarius* |
|  |  |  | *Stomoxys calcitrans* |
|  |  | Mycetophilidae | *Leia fascipennis* |
|  |  | Opomyzidae | *Geomyza tripunctata* |
|  |  | Psilidae | Psilidae sp. 1 |
|  |  |  | Psilidae sp. 2 |
|  |  | Rhagionidae | *Chrysopilus asiliformis* |
|  |  | Rhiniidae | *Stomorhina lunata* |
|  |  | Sarcophagidae | *Sarcophaga agnata* |
|  |  |  | *Sarcophaga carnaria* |
|  |  |  | *Sarcophaga incisilobata* |
|  |  |  | *Sarcophaga subvicina* |
|  |  |  | *Sarcophaga* sp. |
|  |  | Scathophagidae | *Scathophaga stercoraria* |
|  |  | Sciaridae | *Bradysia trivittata* |
|  |  | Stratiomyidae | *Sargus bipunctatus* |
|  |  | Syrphidae | *Dasysyrphus albostriatus* |
|  |  |  | *Episyrphus balteatus* |
|  |  |  | *Eristalis pertinax* |
|  |  |  | *Eupeodes lapponicus* |
|  |  |  | *Melanostoma mellinum/scalare* |
|  |  |  | *Melanostoma scalare* |
|  |  |  | *Meliscaeva auricollis* |
|  |  |  | *Meliscaeva cinctella* |
|  |  |  | *Parasyrphus punctulatus* |
|  |  |  | *Platycheirus scutatus* |
|  |  |  | *Syrphus ribesii* |
|  |  |  | *Syrphus torvus* |
|  |  |  | *Syrphus vitripennis* |
|  |  |  | Syrphidae sp. |
|  |  | Tabanidae | *Atylotus loewianus* |
|  |  |  | *Tabanus bromius* |
|  |  | Tachinidae | *Allophorocera ferruginea* |
|  |  |  | *Blepharomyia pagana* |
|  |  |  | *Blondelia nigripes* |
|  |  |  | *Cyzenis albicans* |
|  |  |  | *Eloceria delecta* |
|  |  |  | *Kirbya moerens* |
|  |  |  | *Linnaemya tessellans* |
|  |  |  | *Loewia foeda* |
|  |  |  | *Lypha dubia* |
|  |  |  | *Ocytata pallipes* |
|  |  |  | *Oswaldia muscaria* |
|  |  |  | *Pales pavida* |
|  |  |  | *Phorocera assimilis* |
|  |  |  | *Phryno vetula* |
|  |  |  | *Phryxe vulgaris* |
|  |  |  | *Pseudoperichaeta nigrolineata* |
|  |  |  | *Ramonda spathulata* |
|  |  |  | *Siphona geniculata* |
|  |  |  | *Triarthria setipennis* |
|  |  |  | *Voria ruralis* |
|  |  |  | *Zaira cinerea* |
|  |  |  | Tachinidae sp. 1 |
|  |  |  | Tachinidae sp. 2 |
|  |  | Tephritidae | *Anomoia purmunda* |
|  |  | Tipulidae | *Nephrotoma appendiculata* |
|  |  |  | *Nephrotoma scalaris* |
|  |  |  | *Tipula fascipennis* |
|  |  |  | *Tipula flavolineata* |
|  |  |  | *Tipula fulvipennis* |
|  |  |  | *Tipula helvola* |
|  |  |  | *Tipula* cf. *hortorum* |
|  |  |  | *Tipula lateralis* |
|  |  |  | *Tipula* cf. *limbata* |
|  |  |  | *Tipula luna* |
|  |  |  | *Tipula lunata* |
|  |  |  | *Tipula* cf. *magnicauda* |
|  |  |  | *Tipula maxima* |
|  |  |  | *Tipula oleracea*/*recondita* |
|  |  |  | *Tipula pagana* |
|  |  |  | *Tipula peliostigma* |
|  |  |  | *Tipula scripta* |
|  |  |  | *Tipula submarmorata* |
|  |  |  | *Tipula* cf. *truncorum* |
|  |  |  | *Tipula vernalis* |
|  |  |  | *Tipula* sp. 1 |
|  |  |  | *Tipula* sp. 2 |
|  |  |  | *Tipula* sp. 3 |
|  |  | Unknown | Diptera sp. 1 |
|  |  |  | Diptera sp. 2 |
|  |  |  | Diptera sp. 3 |
|  |  |  | Diptera sp. 4 |
|  |  |  | Diptera sp. 5 |
|  |  |  | Diptera sp. 6 |
|  |  |  | Diptera sp. 7 |
|  |  |  | Diptera sp. 8 |
|  | Hemiptera | Aphididae | *Tuberculatus querceus* |
|  |  | Aphrophoridae | *Aphrophora alni* |
|  |  | Cercopidae | *Philaenus spumarius* |
|  |  | Cicadellidae | Cicadellidae sp. |
|  |  |  | *Iassus lanio* |
|  |  | Miridae | *Adelphocoris lineolatus* |
|  |  |  | *Lygus* cf. *pratensis* |
|  |  |  | *Neolygus viridis* |
|  |  |  | *Orthotylus prasinus* |
|  |  |  | *Orthotylus viridinervis* |
|  |  |  | *Phytocoris dimidiatus* |
|  |  |  | *Phytocoris longipennis* |
|  |  |  | *Phytocoris tiliae* |
|  | Hymenoptera | Braconidae | *Charmon* sp. |
|  |  |  | *Microplitis* sp. 1 |
|  |  |  | *Microplitis* sp. 2 |
|  |  | Cynipidae | *Andricus quercusradicis* |
|  |  | Ichneumonidae | Ichneumonidae sp. 1 |
|  |  |  | Ichneumonidae sp. 2 |
|  |  | Perilampidae | Perilampidae sp. |
|  |  | Pteromalidae | Pteromalidae sp. |
|  |  | Tenthredinidae | *Periclista pubescens* |
|  |  | Vespidae | *Polistes dominula* |
|  | Lepidoptera | Adelidae | *Nematopogon swammerdamella* |
|  |  | Chimabachidae | *Diurnea fagella* |
|  |  | Depressariidae | *Carcina quercana* |
|  |  |  | *Depressaria douglasella* |
|  |  | Drepanidae | *Cymatophorina diluta* |
|  |  |  | *Polyploca ridens* |
|  |  |  | *Tethea ocularis* |
|  |  |  | *Thyatira batis* |
|  |  | Erebidae | *Catocala nupta* |
|  |  |  | *Catocala promissa* |
|  |  |  | *Catocala sponsa* |
|  |  |  | *Diaphora mendica* |
|  |  |  | *Herminia tarsicrinalis* |
|  |  |  | *Hypena proboscidalis* |
|  |  |  | *Laspeyria flexula* |
|  |  |  | *Phragmatobia fuliginosa* |
|  |  |  | *Polypogon plumigeralis* |
|  |  |  | *Polypogon strigilata* |
|  |  |  | *Rivula sericealis* |
|  |  |  | *Scoliopteryx libatrix* |
|  |  |  | *Spilarctia lutea* |
|  |  |  | *Spilosoma lubricipeda* |
|  |  |  | *Trisateles emortualis* |
|  |  | Gelechiidae | *Bryotropha terrella* |
|  |  |  | *Gelechia turpella* |
|  |  | Geometridae | *Agriopis leucophaearia* |
|  |  |  | *Agriopis marginaria* |
|  |  |  | *Alcis repandata* |
|  |  |  | *Apocheima hispidaria* |
|  |  |  | *Cabera pusaria* |
|  |  |  | *Campaea margaritata* |
|  |  |  | *Camptogramma bilineata* |
|  |  |  | *Chiasmia clathrata* |
|  |  |  | *Chlorissa viridata* |
|  |  |  | *Colotois pennaria* |
|  |  |  | *Crocallis elinguaria* |
|  |  |  | *Cyclophora punctaria* |
|  |  |  | *Deileptenia ribeata* |
|  |  |  | *Dysstroma truncata* |
|  |  |  | *Ectropis crepuscularia* |
|  |  |  | *Ennomos quercinaria* |
|  |  |  | *Epirrhoe alternata* |
|  |  |  | *Epirrita christyi* |
|  |  |  | *Erannis defoliaria* |
|  |  |  | *Eupithecia abbreviata* |
|  |  |  | *Eupithecia dodoneata* |
|  |  |  | *Eupithecia haworthiata* |
|  |  |  | *Eupithecia tantillaria* |
|  |  |  | *Hemithea aestivaria* |
|  |  |  | *Hydriomena furcata* |
|  |  |  | *Idaea aversata* |
|  |  |  | *Idaea fuscovenosa* |
|  |  |  | *Idaea rusticata* |
|  |  |  | *Idaea straminata* |
|  |  |  | *Jodis lactearia* |
|  |  |  | *Lomographa temerata* |
|  |  |  | *Lycia hirtaria* |
|  |  |  | *Macaria liturata* |
|  |  |  | *Odontopera bidentata* |
|  |  |  | *Operophtera brumata* |
|  |  |  | *Operophtera fagata* |
|  |  |  | *Opisthograptis luteolata* |
|  |  |  | *Pasiphila chloerata* |
|  |  |  | *Pasiphila rectangulata* |
|  |  |  | *Pelurga comitata* |
|  |  |  | *Peribatodes rhomboidaria* |
|  |  |  | *Peribatodes secundaria* |
|  |  |  | *Rhodometra sacraria* |
|  |  |  | *Scopula immutata* |
|  |  |  | *Selenia tetralunaria* |
|  |  |  | *Timandra comae* |
|  |  |  | *Xanthorhoe ferrugata* |
|  |  | Hepialidae | *Korscheltellus lupulinus* |
|  |  |  | *Triodia sylvina* |
|  |  | Lasiocampidae | *Lasiocampa quercus* |
|  |  |  | *Malacosoma neustria* |
|  |  | Limacodidae | *Apoda limacodes* |
|  |  | Noctuidae | *Abrostola triplasia* |
|  |  |  | *Acronicta rumicis* |
|  |  |  | *Actinotia polyodon* |
|  |  |  | *Agrochola circellaris* |
|  |  |  | *Agrochola litura* |
|  |  |  | *Agrochola lota* |
|  |  |  | *Agrochola lunosa* |
|  |  |  | *Agrochola lychnidis* |
|  |  |  | *Agrochola pistacinoides* |
|  |  |  | *Agrotis exclamationis* |
|  |  |  | *Agrotis ipsilon* |
|  |  |  | *Agrotis segetum* |
|  |  |  | *Allophyes oxyacanthae* |
|  |  |  | *Amphipyra berbera* |
|  |  |  | *Amphipyra pyramidea* |
|  |  |  | *Anarta trifolii* |
|  |  |  | *Anorthoa munda* |
|  |  |  | *Apamea epomidion* |
|  |  |  | *Apamea lithoxylaea* |
|  |  |  | *Apamea monoglypha* |
|  |  |  | *Apamea scolopacina* |
|  |  |  | *Apamea sordens* |
|  |  |  | *Aporophyla lueneburgensis* |
|  |  |  | *Asteroscopus sphinx* |
|  |  |  | *Atethmia centrago* |
|  |  |  | *Autographa gamma* |
|  |  |  | *Autographa pulchrina* |
|  |  |  | *Axylia putris* |
|  |  |  | *Caradrina clavipalpis* |
|  |  |  | *Charanyca trigrammica* |
|  |  |  | *Conistra erythrocephala* |
|  |  |  | *Conistra rubiginea* |
|  |  |  | *Conistra rubiginosa* |
|  |  |  | *Conistra vaccinii* |
|  |  |  | *Cosmia affinis* |
|  |  |  | *Cosmia pyralina* |
|  |  |  | *Cosmia trapezina* |
|  |  |  | *Craniophora ligustri* |
|  |  |  | *Cryphia algae* |
|  |  |  | *Cucullia umbratica* |
|  |  |  | *Diachrysia stenochrysis* |
|  |  |  | *Dichonia aprilina* |
|  |  |  | *Dicycla oo* |
|  |  |  | *Dryobotodes eremita* |
|  |  |  | *Egira conspicillaris* |
|  |  |  | *Eugnorisma depuncta* |
|  |  |  | *Eugnorisma glareosa* |
|  |  |  | *Eupsilia transversa* |
|  |  |  | *Hadena compta* |
|  |  |  | *Hecatera bicolorata* |
|  |  |  | *Hecatera dysodea* |
|  |  |  | *Helicoverpa armigera* |
|  |  |  | *Heliothis peltigera* |
|  |  |  | *Hoplodrina ambigua* |
|  |  |  | *Hoplodrina blanda* |
|  |  |  | *Hoplodrina octogenaria* |
|  |  |  | *Lacanobia oleracea* |
|  |  |  | *Lacanobia suasa* |
|  |  |  | *Lithophane ornitopus* |
|  |  |  | *Lithophane semibrunnea* |
|  |  |  | *Lithophane socia* |
|  |  |  | *Macdunnoughia confusa* |
|  |  |  | *Mamestra brassicae* |
|  |  |  | *Melanchra persicariae* |
|  |  |  | *Mesapamea secalis*/*secalella*/*didyma* |
|  |  |  | *Mesoligia furuncula* |
|  |  |  | *Mniotype satura* |
|  |  |  | *Mormo maura* |
|  |  |  | *Mythimna albipuncta* |
|  |  |  | *Mythimna conigera* |
|  |  |  | *Mythimna impura* |
|  |  |  | *Mythimna l-album* |
|  |  |  | *Mythimna loreyi* |
|  |  |  | *Mythimna pallens* |
|  |  |  | *Mythimna unipuncta* |
|  |  |  | *Mythimna vitellina* |
|  |  |  | *Noctua comes* |
|  |  |  | *Noctua fimbriata* |
|  |  |  | *Noctua janthe* |
|  |  |  | *Noctua janthina* |
|  |  |  | *Noctua pronuba* |
|  |  |  | *Ochropleura plecta* |
|  |  |  | *Oligia latruncula* |
|  |  |  | *Oligia strigilis* |
|  |  |  | *Oligia versicolor* |
|  |  |  | *Orthosia cerasi* |
|  |  |  | *Orthosia gothica* |
|  |  |  | *Orthosia gracilis*/*opima* |
|  |  |  | *Parastichtis ypsillon* |
|  |  |  | *Peridroma saucia* |
|  |  |  | *Phlogophora meticulosa* |
|  |  |  | *Polia nebulosa* |
|  |  |  | *Polyphaenis sericata* |
|  |  |  | *Pyrrhia umbra* |
|  |  |  | *Spodoptera exigua* |
|  |  |  | *Thalpophila matura* |
|  |  |  | *Tholera decimalis* |
|  |  |  | *Tiliacea aurago* |
|  |  |  | *Tiliacea citrago* |
|  |  |  | *Trachea atriplicis* |
|  |  |  | *Trichoplusia ni* |
|  |  |  | *Xanthia gilvago* |
|  |  |  | *Xanthia icteritia* |
|  |  |  | *Xanthia ocellaris* |
|  |  |  | *Xestia baja* |
|  |  |  | *Xestia c-nigrum* |
|  |  |  | *Xestia rhomboidea* |
|  |  |  | *Xestia xanthographa* |
|  |  |  | Noctuidae sp. 1 |
|  |  |  | Noctuidae sp. 2 |
|  |  |  | Noctuidae sp. 3 |
|  |  |  | *Xylena* sp. |
|  |  | Nolidae | *Bena bicolorana* |
|  |  |  | *Meganola strigula* |
|  |  |  | *Pseudoips prasinana* |
|  |  | Notodontidae | *Drymonia dodonaea* |
|  |  |  | *Drymonia ruficornis* |
|  |  |  | *Harpyia milhauseri* |
|  |  |  | *Phalera bucephala* |
|  |  |  | *Pheosia tremula* |
|  |  |  | *Stauropus fagi* |
|  |  |  | *Thaumetopoea processionea* |
|  |  | Oecophoridae | *Hofmannophila pseudospretella* |
|  |  | Praydidae | *Prays fraxinella* |
|  |  | Pterophoridae | *Emmelina monodactyla* |
|  |  | Pyralidae | *Acrobasis advenella* |
|  |  |  | *Acrobasis repandana* |
|  |  |  | *Acrobasis tumidana* |
|  |  |  | *Agriphila geniculea* |
|  |  |  | *Agriphila inquinatella* |
|  |  |  | *Agriphila selasella* |
|  |  |  | *Agriphila straminella* |
|  |  |  | *Agriphila tristella* |
|  |  |  | *Anania coronata* |
|  |  |  | *Aphomia sociella* |
|  |  |  | *Calamotropha paludella* |
|  |  |  | *Catoptria verellus* |
|  |  |  | *Crambus perlella* |
|  |  |  | *Cydalima perspectalis* |
|  |  |  | *Dioryctria abietella* |
|  |  |  | *Endotricha flammealis* |
|  |  |  | *Eudonia delunella* |
|  |  |  | *Eudonia lacustrata* |
|  |  |  | *Eudonia mercurella* |
|  |  |  | *Galleria mellonella* |
|  |  |  | *Hypsopygia costalis* |
|  |  |  | *Nephopterix angustella* |
|  |  |  | *Nomophila noctuella* |
|  |  |  | *Oncocera semirubella* |
|  |  |  | *Ostrinia nubilalis* |
|  |  |  | *Pediasia contaminella* |
|  |  |  | *Phycita roborella* |
|  |  |  | *Pleuroptya ruralis* |
|  |  |  | *Pyralis farinalis* |
|  |  |  | *Pyrausta despicata* |
|  |  |  | *Pyrausta purpuralis* |
|  |  |  | *Scoparia ambigualis*/*basistrigalis* |
|  |  |  | *Scoparia pyralella* |
|  |  |  | *Sitochroa palealis* |
|  |  |  | *Sitochroa verticalis* |
|  |  |  | *Synaphe punctalis* |
|  |  |  | Pyralidae sp. |
|  |  | Sphingidae | *Deilephila porcellus* |
|  |  |  | *Laothoe populi* |
|  |  |  | *Macroglossum stellatarum* |
|  |  |  | *Mimas tiliae* |
|  |  | Tortricidae | *Acleris cristana* |
|  |  |  | *Archips crataegana* |
|  |  |  | *Archips xylosteana* |
|  |  |  | *Celypha striana* |
|  |  |  | *Cnephasia asseclana* |
|  |  |  | *Cnephasia communana* |
|  |  |  | *Cnephasia cupressivorana* |
|  |  |  | *Cnephasia incertana* |
|  |  |  | *Cnephasia stephensiana* |
|  |  |  | *Cydia fagiglandana* |
|  |  |  | *Cydia pomonella* |
|  |  |  | *Cydia splendana* |
|  |  |  | *Epinotia tenerana* |
|  |  |  | *Eudemis porphyrana* |
|  |  |  | *Eudemis profundana* |
|  |  |  | *Gypsonoma dealbana* |
|  |  |  | *Pandemis cerasana* |
|  |  |  | *Tortrix viridana* |
|  |  |  | *Zeiraphera griseana* |
|  |  |  | *Zeiraphera isertana* |
|  |  | Yponomeutidae | *Yponomeuta* sp. |
|  |  | Ypsolophidae | *Ypsolopha parenthesella* |
|  |  | Unknown | Lepidoptera sp. 1 |
|  |  |  | Lepidoptera sp. 2 |
|  |  |  | Lepidoptera sp. 3 |
|  |  |  | Lepidoptera sp. 4 |
|  | Mecoptera | Panorpidae | *Panorpa germanica* |
|  | Neuroptera | Chrysopidae | *Chrysoperla carnea* |
|  |  |  | *Chrysotropia ciliata* |
|  |  |  | *Nineta flava* |
|  |  |  | *Nothochrysa capitata* |
|  |  |  | *Nothochrysa fulviceps* |
|  |  |  | *Peyerimhoffina gracilis* |
|  |  |  | *Pseudomallada flavifrons* |
|  |  |  | Chrysopidae sp. |
|  |  | Hemerobiidae | *Drepanepteryx phalaenoides* |
|  |  |  | *Hemerobius humulinus* |
|  |  |  | *Hemerobius micans* |
|  |  |  | *Hemerobius pini* |
|  | Orthoptera | Acrididae | *Chorthippus parallelus* |
|  |  | Tettigoniidae | *Leptophyes punctatissima* |
|  |  |  | *Meconema thalassinum* |
|  | Psocodea | Caeciliusidae | *Caecilius fuscopterus* |
|  |  |  | *Valenzuela flavidus* |
|  |  | Psocidae | *Metylophorus nebulosus* |
|  |  |  | *Psococerastis gibbosa* |
|  | Raphidioptera | Raphidiidae | *Subilla confinis* |
|  | Trichoptera | Limnephilidae | *Stenophylax mitis* |
|  |  | Polycentropodidae | *Plectrocnemia conspersa* |
|  |  | Rhyacophilidae | *Rhyacophila dorsalis* |
| Malacostraca | Isopoda | Oniscidae | *Oniscus asellus* |
|  |  | Porcellionidae | *Porcellio scaber* |
|  |  |  | *Porcellio spinicornis* |
|  |  | Unknown | Isopoda sp. |
