# Supplementary material for: The menu varies with metabarcoding practices: A case study with the bat Plecotus auritus
Source: PLoS One. 2019 Jul 5;14(7):e0219135. doi: 10.1371/journal.pone.0219135 (PMC6611578; doi:10.1371/journal.pone.0219135)

# S1 File. Seasonal trophic niche breadth variation (Shannon-Wiener index) measured in *P. auritus*.

The four panels correspond to different data manipulations: A) full dataset, with all prey items kept and identified to the species level and considered as weighted occurrence data (wPOO); B) all unique occurrences discarded from the dataset (No rare items); C) prey identified to the family level only (Family level); D) all prey items weighted according to their relative read abundance (RRA). Significant differences are indicated by a star (P < 0.05).


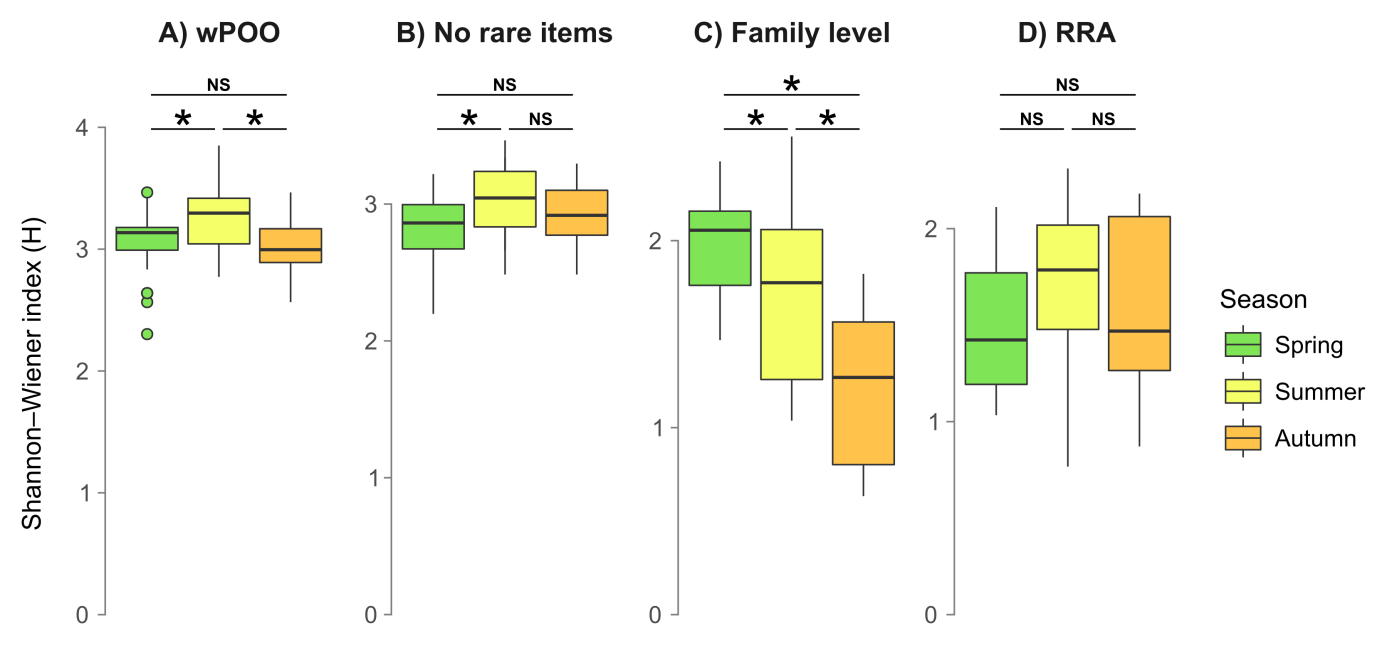

Supplement: S1 File — (DOCX) [file pone.0219135.s002.docx]
