# Supplementary material for: The menu varies with metabarcoding practices: A case study with the bat Plecotus auritus
Source: PLoS One. 2019 Jul 5;14(7):e0219135. doi: 10.1371/journal.pone.0219135 (PMC6611578; doi:10.1371/journal.pone.0219135)

# S2 File. Multidimensional-scaling of trophic niche overlap (Pianka’s O_jk_ index) measured between fecal samples of *P. auritus* from different colonies and collected in distinct seasons.

Each colony is represented by a distinct shape, and seasons by different colours. Size of symbols corresponds to their relative position along the third MDS axis. The four panels correspond to different data manipulations: A) full dataset, with all prey items kept and identified to the species level and considered as weighted occurrence data (wPOO); B) all unique occurrences discarded from the dataset (No rare items); C) prey identified to the family level only (Family level); D) all prey items weighted according to their relative read abundance (RRA).


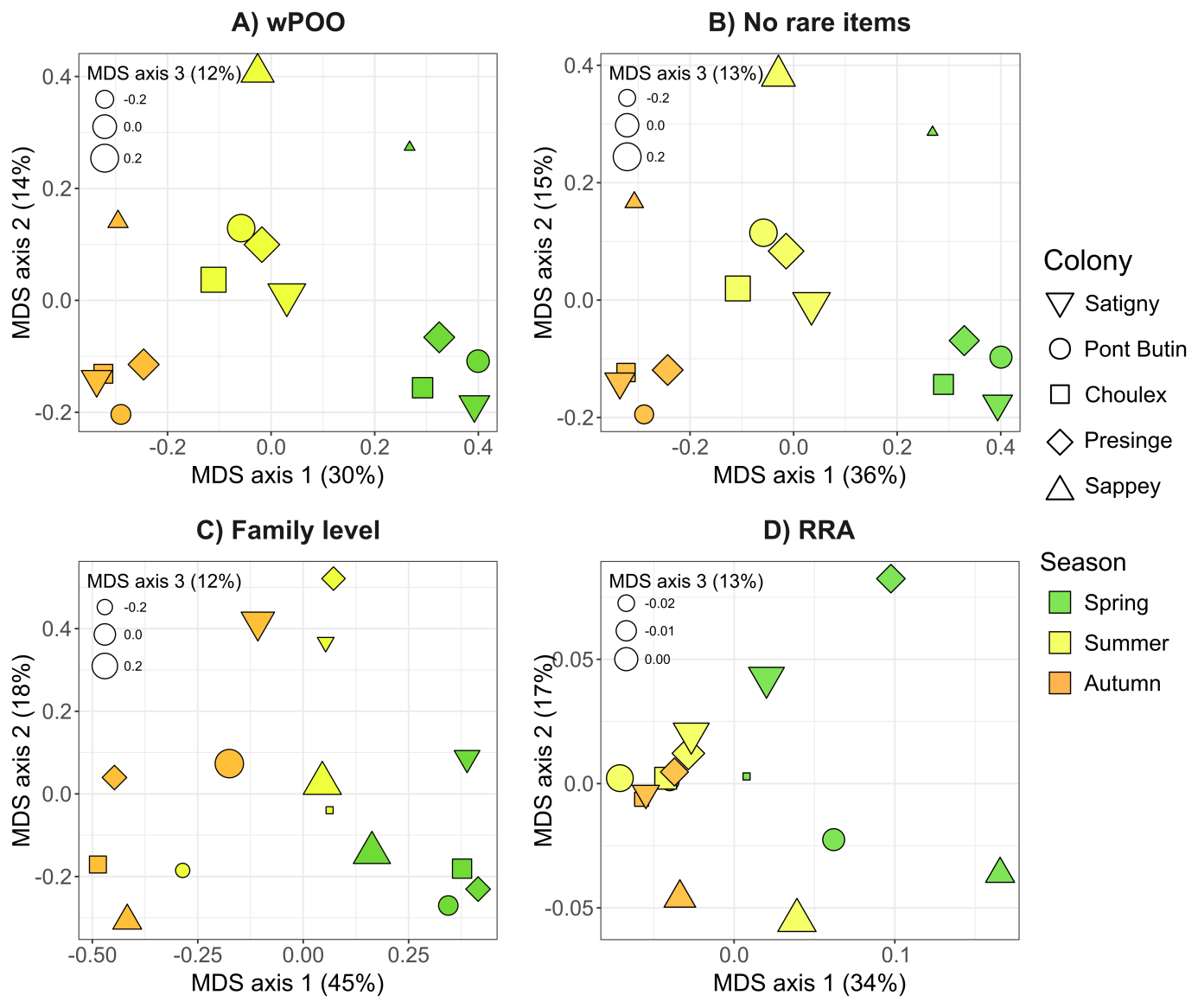

Supplement: S2 File — (DOCX) [file pone.0219135.s003.docx]
