# Supplementary material for: The menu varies with metabarcoding practices: A case study with the bat Plecotus auritus
Source: PLoS One. 2019 Jul 5;14(7):e0219135. doi: 10.1371/journal.pone.0219135 (PMC6611578; doi:10.1371/journal.pone.0219135)
Supplement: S3 File — (DOCX) [file pone.0219135.s004.docx]

# S3 File. Impact of read numbers on frequency estimates.

Lepidoptera sequences were the most common ones when calculating frequency estimates with relative read abundance (RRA), while they were consistently less frequent, though still predominant, in those calculated from weighted percent of occurrences (wPOO) (S3 File - Figure 1). wPOO also underestimated the frequency of beetle sequences when compared to RRA, while dipterans and a number of orders represented by few species (spiders, caddisflies, hymenopterans, cockroaches) were systematically overestimated in wPOO when compared to RRA. Neuropterans were also given more weight in most wPOO approaches, except in one sample (summer, “Sappey”) for which reads number provided a higher frequency than weighted percent of occurrence (6.5 vs 18.3%).


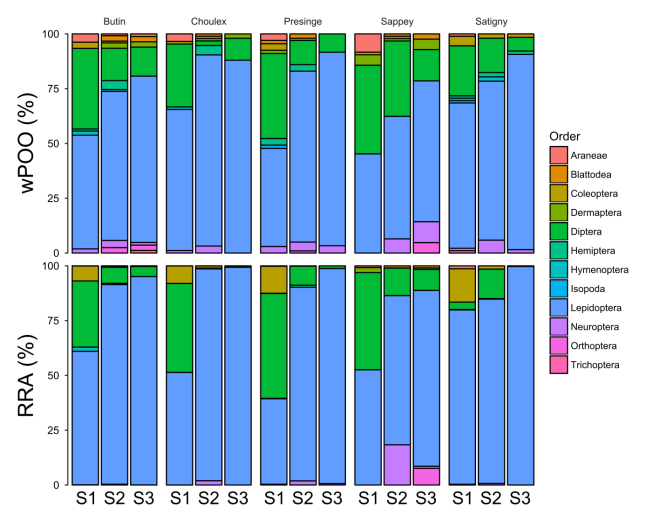


**S3 File - Figure 1.** Relative importance of taxonomic orders in seasonal samples for each colony (S1 – spring, S2 – summer, S3 – autumn).

The effect of weighting prey by read abundance was much higher at the MOTU identification level, as no prey had abundance greater than 6% with wPOO estimates, while RRA identified 8% of prey at least this abundant (and with a RRA score up to 57%) (S3 File - Figure 2). Conversely, half of MOTUs had RRA abundance lower than 0.1%, while none were in this situation with wPOO estimates (lowest RRA score was 0.8%).


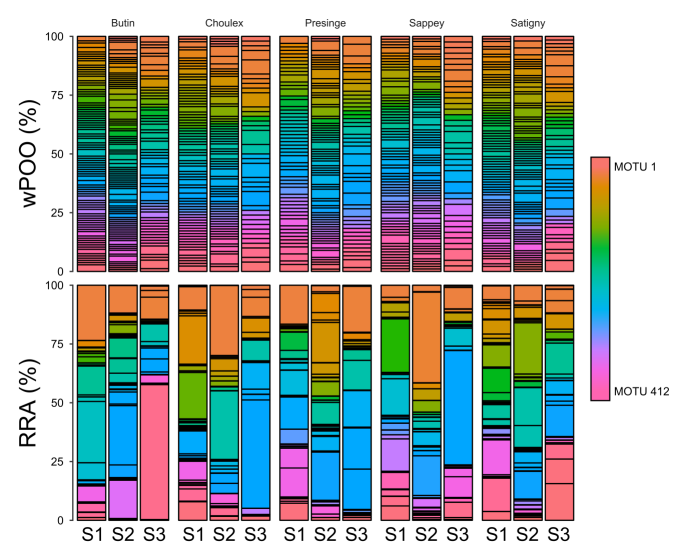


**S3 File - Figure 2.** Relative importance of MOTUs in seasonal samples for each colony (S1 – spring, S2 – summer, S3 – autumn).
